# Neoadjuvant therapy for hepatocellular carcinoma—priming precision innovations to transform HCC treatment

**DOI:** 10.3389/fsurg.2025.1531852

**Published:** 2025-03-06

**Authors:** Kristin E. Goodsell, Alice J. Tao, James O. Park

**Affiliations:** ^1^ Department of Surgery, University of Washington, Seattle, WA, United States; ^2^ Department of Surgery, Mount Sinai Hospital, New York, NY, United States

**Keywords:** neoadjuvant therapy, hepatocellular carcinoma, unresectable hepatocellular carcinoma, immunotherapy, targeted therapy

## Abstract

Hepatocellular carcinoma (HCC) is increasing in prevalence globally, and cure remains limited with non-operative treatment. Surgical intervention, through resection or transplantation, offers a potential for cure for select patients. However, many patients present with advanced or unresectable disease, and recurrence rates remain high. Recent advances in systemic therapies, particularly immune checkpoint inhibitors, have demonstrated promise in treating unresectable HCC and as adjuvant therapy. Evidence from adjuvant trials highlights the synergistic potential of combined liver-directed and systemic therapies. These findings have ignited growing interest in neoadjuvant therapy across various scenarios: (1) as a bridging strategy while awaiting transplantation, (2) for downstaging disease to enable transplantation, (3) for converting unresectable disease to a resectable state, or (4) as neoadjuvant treatment in operable cases. Early-stage trials of neoadjuvant therapy in resectable HCC have reported promising outcomes. To realize the potential of neoadjuvant treatment for HCC, thoughtfully designed, adequately powered, multi-center clinical trials are essential.

## Introduction

Liver cancer is the sixth most common cancer and the third leading cause of cancer-related death globally (1). Hepatocellular carcinoma (HCC), the most prevalent primary liver cancer, typically arises in patients with chronic viral infections, alcohol-related cirrhosis, or metabolic dysfunction associated steatohepatitis. Current treatments prioritize resection or ablation for early-stage disease and transplantation in suitable candidates, with liver-directed or systemic therapies reserved for more advanced disease (2). While surgical resection and transplantation offer potential curative options, overall mortality remains high due to late-stage presentation and frequent recurrence. Notably, the 5-year survival rate for HCC across all stages is approximately 20%, despite the availability of multimodal therapies (3).

The first systemic therapy for HCC, the tyrosine kinase inhibitor (TKI) sorafenib, demonstrated only modest improvement in disease progression and overall survival in advanced disease (4). Despite its limitations, sorafenib remained the only standard treatment until the advent of additional TKIs. However, as an immunogenic cancer, HCC holds great promise for immune-based therapies, such as immune checkpoint inhibitors (ICIs). Recently, the combination of anti-PD-L1 antibody atezolizumab and the VEGF inhibitor bevacizumab (atezo/bev) significantly improved outcomes in unresectable disease compared to sorafenib (5). As these novel therapies emerge as first-line systemic regimens for advanced disease, their potential application in adjuvant and neoadjuvant settings is anticipated.

In this review, we explore the rationale for neoadjuvant therapy in HCC, including its potential roles in bridging patients to transplantation, downstaging disease to enable transplantation, converting unresectable disease, and improving postoperative outcomes. We examine existing treatment modalities for HCC and the synergistic potential of strategic therapy combinations. Finally, we discuss the growing evidence supporting immune checkpoint inhibition in HCC, specifically focusing on promising data for its use in the neoadjuvant setting.

## Rationale for neoadjuvant therapy

The surgical treatment of HCC includes tumor resection or liver transplantation for patients meeting criteria, historically defined by the Milan criteria (6). Neoadjuvant therapy is not currently part of standard treatment for any stage of disease; however, its application could address critical gaps in care (2, 7–9). Advances in systemic therapies highlight opportunities for neoadjuvant treatment to play a transformative role in the following scenarios: (1) bridging to transplantation, (2) downstaging disease to meet transplantation criteria, (3) converting unresectable disease, and (4) improving outcomes in resectable HCC.

### Bridging to transplantation

Patients meeting transplant criteria remain at risk of disease progression while awaiting transplantation (10). Various bridging therapies have demonstrated efficacy in reducing waitlist dropout rates though outcomes have been inconsistent due to the lack of randomized controlled trials (10–19). A systematic review and meta-analysis suggested trends toward improved waitlist retention and post-transplant outcomes with bridging therapy (20). Future efforts should focus on integrating novel and emerging therapies into bridging protocols not only to mitigate waitlist dropout but also to enhance post-transplant survival.

### Downstaging to transplantation

For patients who exceed the Milan criteria for transplant, liver-directed therapies remain a cornerstone for attaining downstaging exception (21). While historically reliant on liver-directed approaches such as transarterial chemoembolization (TACE) or transarterial radioembolization (TARE), recent studies indicate promising outcomes with regimens incorporating ICIs and TKIs (22–24). These approaches not only improve transplant eligibility but may also enhance post-transplant survival, suggesting added therapeutic benefit beyond traditional liver-directed methods (11, 20, 22–30).

### Conversion of unresectable disease

In other malignancies, such as colorectal liver metastases, neoadjuvant systemic therapies have achieved conversion rates up to 60% in phase II trials (31–34). Similarly, evidence suggests that a subset of patients with initially unresectable HCC may achieve conversion with combinations of systemic and liver-directed therapies including chemoradiation (35–38). Recent immunotherapy trials have demonstrated major pathologic responses in resectable HCC, supporting the potential for these strategies to expand the pool of patients eligible for curative surgery (39–41).

### Improved outcomes in resectable disease

Recurrence rates following resection remain high, particularly in high-risk patients such as those with multinodular disease or portal vein tumor thrombus. A 2018 review reported 5-year survival rates for resected multinodular HCC at 35%, dropping to 23% for cases with more than three lesions (42). In other malignancies including pancreatic and colorectal cancer, neoadjuvant therapy is associated with improved outcomes, even for initially resectable disease (43, 44). For patients with HCC, there remains a clear need for improved neoadjuvant therapies for bridging, downstaging and converting disease and also for improving survival in surgical candidates by mitigating the risk of recurrence and metastasis.

## Challenges for systemic therapy in HCC

Hepatocellular carcinoma presents significant challenges in the application of chemotherapy given its low sensitivity to chemotherapeutic agents and multiple mechanisms of chemoresistance (45–47). Single-agent treatments have consistently demonstrated negligible efficacy in HCC, with minimal response rates (47). Sorafenib, a small-molecule inhibitor and a standard systemic treatment for advanced HCC, extends median overall survival (OS) by only 2–3 months, with few partial responses and no complete responses (4, 30). Several mechanisms contribute to HCC's intrinsic “resistome,” enabling resistance to initial chemotherapeutic agents. These mechanisms include dysfunctional DNA repair, downregulation of apoptotic pathways, and the promotion of cancer stem cell survival (45). While combination therapies have demonstrated improved response rates, chemoresistance remains a significant barrier, as initial treatments can induce selective processes that exacerbate resistance, limiting the effectiveness of subsequent adjuvant therapy. Consequently, systemic chemotherapy plays a limited role as first-line treatment outside of advanced disease.

HCC arises commonly from background inflammation and chronic liver disease, which impairs tumor surveillance of the immune microenvironment, dysregulates immune checkpoint interactions, and abrogates T-cell response (48, 49). Overexpression of immune check point molecules programmed death 1 (PD-1), programmed death-ligand 1 (PD-L1), cytotoxic T lymphocyte-associated antigen-4 (CTLA-4) in HCC has been associated with higher recurrence rates, more aggressive disease, and poorer clinical prognosis (50, 51). Immunotherapy with immune checkpoint inhibitors (ICIs) aims to restore effective T-cell responses by targeting these dysregulated pathways.

However, as monotherapies, PD-1 inhibitors (e.g., pembrolizumab), PD-L1 inhibitors (e.g., atezolizumab), and CTLA-4 inhibitors (e.g., tremelimumab) have demonstrated only modest clinical efficacy in HCC (52–55). In the KEYNOTE-240 trial, patients with advanced HCC previously treated with sorafenib who were subsequently treated with either pembrolizumab or placebo had a median progression free survival (PFS) of 3.0 vs. 2.8 months and median OS of 13.9 months compared to 10.6 months (56). Limited efficacy of single-agent ICIs may be attributed to aberrant tumor angiogenesis, which creates a “tumor endothelial barrier” that hinders effective T-cell infiltration of tumor and dampens immune response (57, 58). To address these limitations, combination therapies incorporating agents that inhibit the VEGF pathway (e.g., bevacizumab) which promotes tumor angiogenesis, have been developed to enhance the activity of ICIs (e.g., atezolizumab) and demonstrated improved clinical response (5, 51, 59).

## Evidence for adjuvant therapy

Adjuvant systemic or radiation therapy is a standard component of treatment for many gastrointestinal and hepatobiliary malignancies, including gastric, small bowel, and colon cancer as well as gallbladder cancers and cholangiocarcinoma. Standard adjuvant therapy has been lacking in HCC given historically limited evidence supporting itsefficacy. Other adjuvant therapies aimed to address underlying hepatitis, including vitamin K, cytokine-induced killer cells, and retinoids, have shown improved outcomes in select patients but failed to demonstrate benefits in recurrence free survival (RFS) or OS (2). Although retrospective studies suggested improved postoperative RFS with adjuvant sorafenib, the multi-center phase III STORM trial failed to support these findings (60). Sorafenib demonstrated no improvement over placebo with comparable RFS (33.3 vs. 33.7 months) and OS in patients following resection or ablation (61).

Phase III IMbrave 050 was a highly anticipated adjuvant trial that included patients undergoing resection or ablation with high risk of recurrence (62). Patients were randomized to 12 months of the combination of PD-L1 inhibitor atezo/bev vs. active surveillance. At the first interim analysis with median 17.4-month follow-up, the RFS was significantly better within the treatment vs. active surveillance group (HR 0.72). At the second interim analysis with median 35.1-month follow-up, the difference in RFS was not maintained (33 months for atezo/bev and 36 months for placebo (HR 0.9); therefore, this regimen is not recommended as adjuvant therapy for HCC (63).

However, numerous adjuvant ICI trials are currently underway. These include JUPITER-4 (NCT03859128), a phase II/III trial evaluating anti-PD-1 antibody toripalimab in high-risk patients following resection; KEYNOTE-937 (NCT03867084), a phase III trial of PD-1 inhibitor pembrolizumab vs. placebo in patients with complete radiologic response after resection or ablation; CheckMate 9DX (NCT03383458), a phase III trial investigating adjuvant anti-PD-1 monoclonal antibody nivolumab in high-risk patients; and EMERALD-2 (NCT03847428), a phase III trial examining the adjuvant combination PD-L1 monoclonal antibody durvalumab and bevacizumab compared to durvalumab alone or placebo (64–67).

## Evidence for liver directed therapy

Liver directed therapy is the cornerstone for managing locoregional or unresectable HCC and includes transarterial chemoembolization with or without drug eluting beads (TACE, TACE-DEB), transarterial radioembolization with yttrium-90 microbeads (TARE, Y90), radiofrequency or microwave ablation (RFA, MWA), hepatic artery infusion chemotherapy (HAIC), and external beam/stereotactic body radiation therapy (EBRT/SBRT). These treatments can be administered with curative intent for smaller lesions or used alone or in combination as bridging therapy, downstaging approaches, or for controlling unresectable disease.

### Ablation

Thermal ablation using RFA or MWA is the primary ablative therapy, while percutaneous ethanol injection (PEI) and cryoablation are less frequently used. Thermal ablation is indicated for accessible tumors with normal tissue margin and limited by proximity to critical structures such as blood vessels, bile ducts, or adjacent organs. For smaller lesions, ablation may be performed alone with curative intent, while intermediate or larger lesions may require combination therapy.

In a 2021 meta-analysis, RFA demonstrated comparable disease-free survival (DFS) and OS compared to resection in patients meeting Milan criteria (68). Similarly, the SURF trial found no significant difference in RFS between surgery and RFA for tumors up to 3 cm (69). Interestingly, a more recent meta-analysis indicated that while OS and RFS in tumors <3 cm or > 5 cm favored surgical resection, RFA and resection were equally effective for tumors 3–5 cm (70).

A 2020 proof of concept trial suggested synergistic benefits of RFA during anti-PD-1 therapy, demonstrating more than a two-fold increase in complete and partial response rates (71). This supports the hypothesis that liver-directed therapies may generate neo-antigens, enhancing the efficacy of immunotherapies.

### Embolization

TACE is a mainstay of treatment for intermediate-stage HCC and requires relative isolation of blood supply to vascularized tumor targets. Bland embolization (TAE) induces ischemia and tumor necrosis and has been shown to improve survival compared to supportive care (72). TACE builds on TAE by delivering concentrated chemotherapy in conjunction with inducing ischemia and has demonstrated superior outcomes relative to TAE in unresectable HCC (73). The addition of drug eluting beads (TACE-DEB) may further enhance response rates and has been associated with longer OS in subsets of patients (74, 75). TARE, using yttrium-90 microspheres, delivers tumor targeted radiotherapy and is employed primarily for unresectable HCC (75, 76). Evidence supports that TARE can serve a neoadjuvant role prior to transplant or resection with 3-year OS > 90% in patients undergoing transplant or resection (77).

### Hepatic arterial infusion chemotherapy (HAIC)

HAIC delivers high concentrations of cytotoxic drugs directly to the liver while minimizing systemic side effects. While not a standard therapy, it is recommended in certain guidelines as an alternative treatment for advanced disease (78). In a phase III clinical trial, HAIC with FOLFOX demonstrated superior OS compared to sorafenib (13.9 vs. 8.2 months) in patients with locally advanced HCC (79). Downstaging occurred in 12.3% of patients and those that underwent resection achieved an OS of 20.8 months. Another phase III trial comparing FOLFOX-HAIC to TACE found improved OS (23.1 vs. 16.1 months), PFS (9.6 vs. 5.4 months), and response rate (46% vs. 18%) for HAIC (80). A 2024 meta-analysis further suggested that HAIC combined with ICIs or targeted therapies could yield synergistic effects and produce enhanced outcomes (81).

### Radiotherapy (SBRT)

Although not a first line treatment, SBRT is commonly used for local control and as a bridging therapy in transplant candidates. A recent phase II trial reported response rates of 62.5–78.1% in patients undergoing SBRT as bridge towards transplant (82). In locally advanced HCC, combined chemoradiation and HAIC have achieved downstaging in 78% of patients, highlighting the potential of radiotherapy as part of a multimodal regimen (36).

### Histotripsy

Histotripsy applies short, high amplitude ultrasound pulses to induce cavitation in focal tissues. This nonthermal, nonionizing technique maximizes the targeted advantages of ablative therapy while eliminating limitations such as the “heat sink effect,” off target thermal spread, and thermal fixation (83). The multicenter phase I THERESA trial was the first feasibility study and reported good concordance between tissue destruction and planned target volume without device-related adverse events (84). The prospective, multicenter, single-arm HOPE4LIVER trial also reported technical success with low treatment-related complications (85, 86). Future trials are needed to evaluate the long-term efficacy of this modality as a locoregional therapy.

### Summary of liver-directed therapy

Liver-directed locoregional therapies (LDT/LRT) play a critical role in bridging, downstaging, or converting unresectable disease. While randomized clinical trials are limited, evidence supports their use in bridging patients with projected long wait times (87). Patients downstaged with LRT appear to have comparable outcomes relative to patients meeting Milan Criteria upfront. Yao et al.'s protocol including TACE or RFA/PEI successfully downstaged 65% of patients with no significant difference in 1- and 5-year post-transplant survival compared to patients meeting Milan criteria upfront (93.4/77.8% vs. 94.3/81% *p* = 0.69) (88). Similarly, Chapman et al. reported a >40% downstaging rate with comparable 5-year recurrence (10.9% vs. 10.8%, *p* = 0.84) and post-transplant OS (26). Downstaging therapy with TACE/TARE +/- sorafenib has shown higher risk of recurrence but comparable OS compared to tumors meeting Milan criteria upfront (27). Conversion therapy is an area of active investigation, with emerging evidence suggesting enhanced potential when LRTs are combined with systemic treatments (36, 71, 89–92).

## Evidence for combination therapy

As discussed, multimodal treatment of HCC offers opportunities for therapeutic synergy, particularly through the combination of LDT/LRT with ICIs or targeted therapies (93). For example, TACE induces cellular damage and tumor necrosis, generating neoantigens that can prime the adaptive immune response when paired with ICI. Concurrently, TACE-induced tissue hypoxia stimulates angiogenesis, which can be mitigated by VEGF inhibitors. Similarly, LRTs such as ablation, radiation or histotripsy may generate tumor neoantigens that enhance immune responses or potentiate the effects of targeted therapies.

These combined therapies have primarily been studied in intermediate to advanced and unresectable HCC (71). Interim analysis from the multi-center phase III LAUNCH trial demonstrated improved PFS (10.6 vs. 6.4 months, HR 0.43) and OS (17.8 vs. 11.5 months, HR 0.45) in patients treated with lenvatinib plus TACE vs. lenvatinib alone (94). Several ongoing or recently completed trials are evaluating the impact of combined therapies. The phase III EMERALD-1 trial (NCT03778957) examines TACE and durvalumab, with or without bevacizumab, or TACE alone (95). Interim analysis showed improved PFS for TACE plus durvalumab and bevacizumab vs. TACE alone (15.0 vs. 8.2 months, HR 0.77). The phase III LEAP-012 trial (NCT04246177) evaluates TACE plus lenvatinib and pembrolizumab vs. TACE plus placebo (96). Interim analysis demonstrated improved PFS for the combination (14.6 vs. 10.0 months, HR 0.66) (97). EMERALD-3 (NCT05301842) is a phase III trial comparing TACE plus tremelimumab plus durvalumab +/- lenvatinib vs. TACE monotherapy (98). The phase III CheckMate-74W trial (NCT04340193) sought to compare TACE with nivolumab and ipilimumab vs. nivolumab alone or placebo but was terminated due to slow accrual. The TACE-3 (NCT04268888) phase II/III trial will assess nivolumab in combination with TACE/TAE (99).

Collectively, these findings underscore the potential of multimodal approaches to enhance outcomes in HCC, supporting the concept of synergy between LDTs and systemic therapies and potential use in the neoadjuvant setting.

## Evidence for immune-based neoadjuvant therapy

Several early phase trials have evaluated immune-based therapies in the neoadjuvant setting for HCC, with promising preliminary findings and no significant delays to surgery.

The phase Ib PRIME-HCC trial (NCT03682276) evaluated ipilimumab and nivolumab prior to resection, reporting no delays in surgery due to immune checkpoint inhibition (40, 100). All patients had pathologic responses seen on surgical specimens, with 78% achieving partial and 22% complete responses. A phase Ib/II trial in resectable disease evaluated the safety/efficacy of toripalimab vs. toripalimab plus lenvatinib (NCT03867370) (101). Among patients who proceeded to surgery, 20% had major pathological responses. In the Phase II trial of camrelizumab plus apatinib (NCT04297202) for resectable disease, most patients completed treatment, and no surgical delays were reported (102). More than 20% of patients had a partial or complete pathological response, and circulating tumor DNA (ctDNA) positivity correlated with a lack of major pathological response. In the phase II trial for cemiplimab (NCT03916627) in resectable disease, one patient had surgery delayed due to medication adverse effects, however, there were no grade 4 or 5 adverse effects observed (39). Notably, 35% of patients achieved significant or partial pathologic response. Another phase II trial (NCT03222076) explored neoadjuvant and adjuvant nivolumab plus ipilimumab vs. nivolumab monotherapy in resectable disease (41, 103). No patients experienced delays in surgery due to medication effects. Major pathological response was observed in a subset of patients in both groups (27% nivolumab plus ipilimumab vs. 33% nivolumab), and PFS was higher in the combination group (19.5 vs. 9.4 months).

Whereas most trials for neoadjuvant therapy involve resectable disease, one phase Ib trial evaluated cabozantinib and nivolumab in borderline resectable or locally advanced HCC (NCT03299946) (104). Of the 14 patients treated, 12 underwent successful surgical resection with negative margins. Notably, 25% of patients had a major pathologic response and 1 patient had a complete pathologic response, highlighting the potential for converting borderline resectable or locally advanced cases using neoadjuvant therapy. Enrichment in effector T cells, tertiary lymphoid structures, and distinct B cell and plasma cell profiles was found in responders (105).

Other studies have examined the use of LRTs alone or in conjunction with systemic therapy in the neoadjuvant setting. A retrospective analysis noted that combination of TACE and HAIC vs. TACE alone led to enhanced conversion rates for initially unresectable disease (106). A phase II trial combining lenvatinib, toripalimab and FOLFOX-HAIC in advanced HCC demonstrated response rates exceeding 60% (107).

In summary, these studies underscore the feasibility and safety of neoadjuvant immune-based and combination therapies, with minimal delays to surgery. Notably, initial evidence supports the potential for converting borderline or unresectable HCC to resectable cases. Additionally, biomarkers such as ctDNA. have emerged as valuable tools for monitoring treatment response, detecting disease recurrence or progression, and ultimately informing clinical and surgical decision making. Ongoing and future trials are needed to shed light on optimal strategies for leveraging novel anti-cancer therapies.

## Future directions

Neoadjuvant immunotherapy has the potential to transform the management of HCC. By inducing immediate tumor reduction, expanding effector and memory CD4 + and CD8+ T cell populations, and enhancing interferon-gamma and granzyme B activity, it primes the immune system to eliminate residual micrometastatic disease and reduces the risk of recurrence (39, 51, 104).

Particularly when combined with other treatment modalities, neoadjuvant immunotherapy has shown potential to amplify anti-tumor effects while minimizing toxicity to functional liver parenchyma. Multiple trials are underway to further explore the effects of combination therapy (Table 1), including dual immunotherapies, immunotherapy with anti-VEGF agents, and immunotherapy with LDTs, including HAIC, TACE, TARE, and ablation. LRTs can stimulate the immune response by releasing tumor antigens from tumor debris, potentiating antitumor immune responses and reducing the risk of micrometastatic recurrence (108). The synergistic effects of immunotherapy and locoregional therapy may enable downstaging advanced disease for curative intent resection or bridging to transplant (109). However, many current trials have small cohorts and lack comparison groups, limiting the validation of specific combinations, optimal regimens, and treatment durations.

**Table 1:** Neoadjuvant trials in hepatocellular carcinoma.

National Clinical Trial Number	Study Status	Type	Intervention	Surgery	Primary Outcome Measures	Primary Completion Date (Estimated)
NCT06405321	Recruiting	Retrospective	HAIC vs. TACE vs. TKIs vs. ICIs vs. radiotherapy	SNS	OS (1 year)	December 2024
NCT06003673	Recruiting	Single arm, prospective	Tislelizumab with TACE and Lenvatinib	SR	RFS (up to 36 months)	July 2024
NCT05137899	Recruiting	Two arms, prospective, randomized	Neoadjuvant Atezolizumab/Bevacizumab vs. SBRT	SR	Proportion of patients undergo hepatectomy (17 weeks)	June 2026
NCT05185531	Active, not recruiting	Single arm, prospective	Neoadjuvant Tislelizumab and SBRT	SR	Delay to surgery, ORR, pathological response rate, safety and tolerability	December 2024
NCT05389527	Active, not recruiting	Single arm, prospective	Neoadjuvant lenvatinib + pembrolizumab	SR	MPR (up to 24 weeks)	December 2023
NCT06349317	Recruiting	Single arm, prospective	Neoadjuvant IMRT with perioperative camrelizumab + apatinib	SR	EFS (1 year)	May 2026
NCT03578874	Completed	Single arm, prospective	Neoadjuvant SECOX (sorafenib, capecitabine, oxaliplatin)	SR	Resectability (end of cycle 4)	April 2019
NCT05908786	Recruiting	Three arms, prospective, randomized	Neoadjuvant atezolizumab + bevacizumab vs. atezolizumab + bevacizumab + tiragolumab vs. tobemstomig + bevacizumab	SR	MPR (at time of surgery)	September 2025
NCT04930315	Recruiting	Two arms, prospective, randomized	Neoadjuvant camrelizumab + apatinib mesylate with adjuvant camrelizumab vs. adjuvant camrelizumab	SR	RFR (1 year)	June 2024
NCT06664996	Recruiting	Single arm, prospective	Neoadjuvant SBRT with sintilimab	SR	DFS (1 year)	October 2025
NCT03510871	Completed	Single arm, prospective	Nivolumab + ipilimumab	SR	% patients with tumor shrinkage (4 years)	May 2024
NCT05171335	Enrolling	Single arm, prospective	Lenvatinib with TACE vs. historical control	LT	% tumor necrosis at time of transplant	June 2026
NCT05250843	Recruiting	Two arms, prospective, randomized	Neoadjuvant TACE/HAIC + Lenvatinib + Sintilimab vs. direct surgery	SR	RFS (1 year)	December 2023
NCT05701488	Recruiting	Two arms, prospective, randomized	Neoadjuvant durvalumab + tremelimumab vs. durvalumab + tremelimumab + SIRT	SR	AE (18 months)	October 2025
NCT05225116	Recruiting	Single arm, prospective	Sintilimab + Lenvatinib + Radiotherapy	SR	AE, proceed to surgery (5 years)	December 2025
NCT04857684	Recruiting	Single arm, prospective	Neoadjuvant SBRT + Atezolizumab + Bevacizumab	SR	AE (6 months)	December 2024
NCT05185505	Recruiting	Single arm, prospective	Atezolizumab + Bevacizumab + TACE	LT	Proportion of patients with acute allograft rejection of transplant (1 year)	April 2027
NCT06569498	Recruiting	Single arm, prospective	TACE + lenvatinib + camrelizumab	SR	MPR, AE (1 year), operative mortality (1 month), operative complications (1 year), reoperation rate (1 year)	February 2026
NCT04224480	Active, not recruiting	Single arm, prospective	Neoadjuvant and adjuvant pembrolizumab	SR	Number of patients with HCC recurrence, number of CD8+ Ki67+ T cells found in resected tumor (2 years)	December 2025
NCT04615143	Recruiting	Two arms, prospective, non-randomized	Neoadjuvant tislelizumab vs. tislelizumab + lenvatinib	SR	DFS (1 year)	December 2024
NCT05440864	Recruiting	Single arm, prospective	Neoadjuvant Tremelimumab + Durvalumab and adjuvant Durvalumab	SR	AE (4 years)	November 2025
NCT04424043	Recruiting	Two arms, prospective, randomized	Neoadjuvant TACE-HAIC + Surgery vs. Surgery (BCLC B Stage HCC)	SR	PFS (36 months)	December 2022
NCT04777942	Recruiting	Two arms, prospective, randomized	Neoadjuvant TACE-HAIC + Surgery vs. Surgery (BCLC A Stage HCC)	SR	PFS (36 months)	December 2023
NCT06524466	Active, not recruiting	Single arm, prospective	Neoadjuvant SBRT with Lenvatinib and Pucotenlimab	SR	ORR, TCR (1 year)	December 2026
NCT06492408	Recruiting	Three arms, prospective, randomized	Double ICI vs. double ICI + chemo vs. double ICIs + chemodrug + bevacizumab	SR	PCR, MPR (6 months)	December 2028
NCT05194293	Recruiting	Single arm, prospective	regorafenib + durvalumab	SR	ORR (16 weeks)	December 2024
NCT05613478	Recruiting	Two arms, prospective, randomized	Neoadjuvant TACE + camrelizumab + apatinib mesylate vs. surgery	SR	RFS (3 years)	November 2025
NCT04425226	Recruiting	Two arms, prospective, randomized	Neoadjuvant Pembrolizumab + Lenvatinib vs. none prior to transplant	LT	RFS (4 years)	December 2022
NCT04521153	Active, not Recruiting	Two arms, prospective, randomized	Neoadjuvant Camrelizumab + Apatinib Mesylate vs. surgery	SR	EFS (3 years), MPR (30 days)	July 2025
NCT05920863	Recruiting	Single arm, prospective	Neoadjuvant Lenvatinib + Tislelizumab + TACE	SR	MPR (16 weeks)	May 2025

AE, adverse events; BCLC, Barcelona Clinical Liver Cancer; DFS, disease free survival; EFS, event free survival; LT, liver transplant; MPR, major pathologic response; ORR, objective response rate; PCR, pathologic complete response; PVTT-HCC, portal vein tumor thrombus-hepatocellular carcinoma; RFR, recurrence free rate; SECOX, sorafenib, capecitabine, oxaliplatin; SNS, surgery not specified; SR, surgical resection; TCR, treatment complete rate.

Identifying novel HCC biomarkers may enable early detection, the development of targeted therapy, and the identification of tumor resistance. Recent advances in sequencing technologies and ctDNA detection methods have broadened the potential role of liquid biopsies. ctDNA-based liquid biopsies can identify new biomarkers, measure treatment response, estimate tumor mutational burden, and assess minimal residual disease to predict recurrence risk or need for adjuvant immunotherapy (109–113).

Beyond ICIs, other immune interventions that are being explored include allogenic natural killer (NK) cells, chimeric antigen receptor T (CAR-T) cells, oncolytic viruses, and vaccines (114). The Phase IIb TRAVERSE study investigated a vaccinia virus-based oncolytic immunotherapy (pexastimogene devacirepvec) in patients who progressed on sorafenib. While it did not improve OS compared to best supportive care, it demonstrated induction of a T-cell response (115). The use of CAR-T-cell therapy in solid tumors has so far been limited, but preclinical models have demonstrated potential for further applications which are being explored in clinical trials (116, 117).

## Discussion

Hepatocellular carcinoma is projected to increase in incidence and mortality. The armamentarium for the treatment of unresectable, recurrent, and metastatic disease has expanded. Neoadjuvant and combination therapies have the potential to redefine HCC management, offering improved outcomes for resectable, borderline resectable, and advanced disease. ICIs have shown safety and efficacy in early trials, while combinations with anti-VEGF agents, LRTs like TACE and ablation, and systemic treatments enhance anti-tumor effects while preserving liver function. LRTs stimulate immune responses by generating tumor neoantigens, further boosting the impact of immunotherapy. Emerging biomarkers like ctDNA enable precise monitoring of treatment response and recurrence risk. Novel approaches, including CAR-T cells and oncolytic viruses, are expanding therapeutic possibilities. While current trials face limitations, these advancements highlight the transformative potential of multimodal strategies in improving surgical outcomes, reducing recurrence, and achieving curative results in advanced HCC. Continued research will be vital to optimize and standardize these approaches to change the surgical—and survival—landscape for this deadly disease.
